# Evaluation of Cat Exposure to Bisphenol A (BPA) Using Hair Sample Analysis

**DOI:** 10.3390/ani16040567

**Published:** 2026-02-12

**Authors:** Slawomir Gonkowski, Manolis Tzatzarakis, Elena Vakonaki, Thomas Lamprakis, Krystyna Makowska

**Affiliations:** 1 Department of Clinical Physiology, Faculty of Veterinary Medicine, University of Warmia and Mazury in Olsztyn, Oczapowskiego 13, 10-719 Olsztyn, Poland; 2 Laboratory of Toxicology, School of Medicine, University of Crete, 71003 Heraklion, Crete, Greece; tzatzarakis@uoc.gr (M.T.); evakonaki@gmail.com (E.V.); lamprakist@gmail.com (T.L.); 3 Department of Clinical Diagnostics, Faculty of Veterinary Medicine, University of Warmia and Mazury in Olsztyn, Oczapowskiego 14, 10-719 Olsztyn, Poland

**Keywords:** companion animals, endocrine disruptors, bisphenol A, fur, biomonitoring

## Abstract

Bisphenol A (BPA) is a synthetic organic compound commonly used as a plasticiser in the industry. It pollutes the environment, harms human and animal organisms, and exhibits endocrine-disrupting properties. Companion animals, living in proximity to humans, are highly exposed to BPA. However, knowledge of cat exposure to this compound is extremely scarce. In the present study, BPA levels were analysed for the first time in cat hair using liquid chromatography–triple quadrupole mass spectrometry. BPA concentrations ranged from below the limit of detection to 955.4 pg/mg, with a mean (±standard deviation) of 67.98 ± 145.2 pg/mg and a median of 27.3 pg/mg. Higher levels of BPA have been found in the strictly indoor cats (mean 79.45 ± 162.2 pg/mg, median 35.3 pg/mg) than in cats with outdoor access (mean 25.93 ± 8.07 pg/mg, median 24.4 pg/mg). Some differences in BPA levels have also been noted depending on age and body condition scores. The results show that cats are exposed to BPA to a considerable extent, and the levels of this compound in hair may depend on many factors. It can be assumed that BPA may negatively affect cat health, but due to limited knowledge of BPA metabolism in cats and its harmful effects in this species, many aspects of these issues require further comprehensive studies.

## Simple Summary

Bisphenol A (BPA) is an organic compound that is commonly used in various branches of industry, mainly as a component of plastics. BPA pollutes the environment and harms living organisms. It is known that companion animals living in proximity to humans are exposed to anthropogenic environmental pollutants to a large extent. Still, knowledge of cat exposure to BPA is extremely limited. The study describes, for the first time, BPA levels in cat hair samples, which are the best matrix for assessing long-term exposure to environmental pollutants. The results indicate the presence of BPA in cat hair, suggesting that cats are significantly exposed to this compound. During the study, some relationships between BPA levels in the hair and cat lifestyle, age and body condition scores have been noted. The results show that cats are exposed to BPA, and this substance may negatively affect their health, but further studies are needed to clarify all aspects of its effects on cat health.

## 1. Introduction

Bisphenol A (BPA)—the name preferred by the International Union of Pure and Applied Chemistry (IUPAC) for 4,4′-(Propane-2,2-diyl)diphenol—is an organic substance synthesised for the first time in the late 19th century [1]. This compound is characterised by plasticity, lightness, mechanical resistance and a relatively high melting point [2]. These features, together with its low synthesis cost, have led to BPA’s use in industry since the 1930s [2,3]. BPA is commonly used as a plasticiser, i.e., an additive increasing plasticity in the production of polycarbonate plastics, in which this substance may constitute up to 90% of the mass [4]. BPA is present in many everyday items, such as food containers, bottles, kitchen appliances, electronic or car equipment, home furnishing, clothes and even dental materials [2,3]. BPA may also be an ingredient of paints, varnishes and brake fluid. It is estimated that over 5 billion tonnes of BPA are produced annually [4].

The large-scale production and widespread use of BPA result in substantial amounts of this compound entering the natural environment [3,5]. To date, BPA has been detected in surface waters, groundwater, soil, and the atmosphere worldwide [6,7,8,9]. It is also known that BPA may penetrate human and animal organisms, mainly through the digestive tract with food and drinking water, but also through the respiratory system, the skin, and transplacental transport during the prenatal period [2,3,10]. In living organisms, BPA, due to its structural similarity to oestrogens, has endocrine-disrupting effects [3]. BPA-induced hormonal dysregulation impairs the function of many internal organs [11]. The harmful effects of BPA on the ovaries, testes, uterus, brain, liver, heart, kidneys, and other internal organs have been well documented [3,12,13,14,15]. BPA may impair immune reactions and metabolism processes [16,17,18]. Previous studies have also reported that BPA has neurotoxic, genotoxic, proinflammatory and cancerogenic activities [16,19,20].

Due to its strong, multidirectional toxic properties, monitoring human and animal exposure to BPA is an important task in modern environmental toxicology. Knowledge of human exposure to BPA is relatively extensive. Numerous studies have reported the presence of BPA in various matrices, mainly in blood serum and urine, but also in other body fluids, such as breast milk, saliva, amniotic fluid, and semen plasma [21,22,23,24]. BPA has also been found in human faeces, nails and hair [25,26,27].

Among the above-mentioned matrices, hair is particularly interesting and becoming increasingly important [25,28,29]. This is because, unlike “classic” matrices (such as serum or urine), BPA accumulates in hair and its levels do not change quickly in the short term [30]. Therefore, hair, as one of the few matrices, can be used to assess long-term environmental exposure to BPA, lasting several weeks or even several months [30]. Additionally, previous studies have shown that hair analysis for BPA is comparable in terms of specificity, accuracy and sensitivity to serum and urine analysis [25,29]. Moreover, experimental studies have shown that BPA levels in hair reflect the degree of exposure to this compound and change following exposure [31]. Another advantage of hair as a matrix is that sample collecting is painless and stress-free, which is especially important in research on aggressive and shy animals. Because hair does not decay quickly after the death of the organism, and the levels of substances contained in it do not change, hair samples can also be collected from carcasses. Moreover, hair samples may be easily stored and transported. Nevertheless, to date, hair samples have been used mainly for biomonitoring human exposure to BPA [25,28,29] and much less often for other species [32,33].

**Figure 1:**
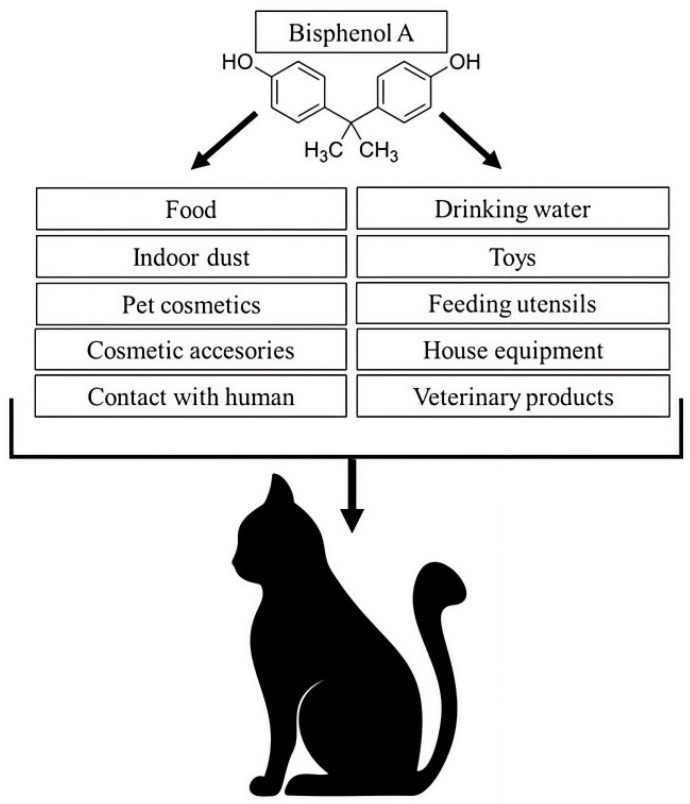
Sources of the exposure to BPA in cats (authors’ own conceptual model).

It should be noted that knowledge of the exposure of companion animals (especially cats) to BPA is relatively limited. Until recently, the role of BPA as a potential animal health hazard has been marginalised in veterinary medicine. However, it is known that dogs and cats are extensively exposed to BPA and other anthropogenic environmental pollutants [34,35]. This is because dogs and cats live in close proximity to humans, in homes, and are therefore exposed to the same environmental factors as humans [36] (Figure 1). Additionally, some studies have reported that, due to their smaller size, companion animals are more exposed than humans to certain factors (such as indoor dust) that affect exposure to BPA and other pollutants [36,37,38]. For this reason, companion animals, especially cats, which usually spend most or all of their lives at home, are considered sentinel species that can warn people about toxic substances in the home environment [37].

Current knowledge about BPA in cats is severely limited and fragmented. Few studies have reported the presence of BPA in cat serum [34], urine [35,39] and faeces [35], as well as in cat commercial food [40,41]. It is also known that BPA affects the cat’s nervous system and uterine muscles [42,43]. However, to date, the long-term exposure of cats to BPA has not been evaluated using hair sample analysis.

Given the above, the present study aimed to examine for the first time the levels of BPA in cat hair samples. The second aim was to determine whether the degree of cat exposure to BPA is associated with gender, age, body condition score, and whether the cat is indoors or outdoors. The results of the present study will increase the understanding of cats’ long-term exposure to BPA and may be the first step toward reducing this exposure and thereby improving cats’ health.

## 2. Materials and Methods

### 2.1. Reagents

The following reagents were used during the study: BPA (≥98%) from Sigma-Aldrich (St. Louis, MO, USA); methanol (LC-MS grade) from Fischer Chemicals (Loughborough, UK); phenobarbital-d5 (internal standard—IS) from Supelco (Bellefonte, PA, USA); and ultrapure water, obtained using a Merck Direct-Q 3UV water purification system (Merck, Darmstadt, Germany).

### 2.2. Sampling

The study included 70 clinically healthy, non-purebred cats of both genders (37 males and 33 females), aged 1 to 15 years. All cats belonged to private owners and were castrated/sterilised. According to information from cat owners, all animals in the experiment were fed similarly, receiving both dry and canned (wet) commercial food intended for this species. All cats included in the study lived in Olsztyn (a city in northeastern Poland, coordinates 53°46′40″ N 20°28′45″ E), whose characteristics are presented in Table 1.

The body condition of the cats included in the study was evaluated using the International Cat Body Condition Score system, in which a BCS of 1–4 indicates too-thin animals, a BCS of 5 indicates animals with correct weight and physiological body condition, and a BCS of 6–9 indicates obese animals [44]. Moreover, all cats included in the study were divided into three age groups: young (≤2 years), adult (≤6 years), and mature and senior (>6 years) cats. This division was made based on the physiology of cat maturation [45]. In this animal species, growth, maturation, and development continue until the age of 2. After this age, the cat’s behaviour and metabolism change significantly. Further changes occur after age 6, when the ageing process begins in the cat’s body. Additionally, according to information obtained from the owners, cats were divided into outdoor and indoor animals. Characterisation of the animals included in the study is presented in the Supplementary Materials (Table S1).

Hair samples (weighing about 2 g) were collected from all animals in October and November 2023 during veterinary and/or care treatments from the same place (the central part of the abdomen). The hair was cut as close to the skin as possible with metal scissors, and the collected hair ranged in length from 0.7 to 1.8 cm. Immediately after collecting hair samples, they were wrapped in aluminium foil. Samples were then stored in a dark place at room temperature until further study. Before sampling, the cats’ owners were informed of the study’s aim and gave verbal consent for their animals’ participation. Because sampling was performed during care and/or veterinary procedures and was completely non-invasive, stress-free, and painless for the animals, Ethics Committee approval was not required. This is in accordance with the law in force in Poland—the Act for the Protection of Animals for Scientific or Educational Purposes of 15 January 2015 (Official Journal of Laws of the Republic of Poland 2015, item 266).

### 2.3. BPA Extraction

All collected hair samples were analysed for BPA using the method previously described by Tzatzarakis et al. [46] and Makowska et al. [47]. The hair was cut with metal scissors into fragments measuring several millimetres. The hair was then rinsed four times (twice in ultrapure water and twice in methanol) to remove the external contaminations from the hair surface. After rinsing, the hair was dried at 50 °C and 100 mg of each sample was extracted with 2 × 2 mL of methanol and 25 ng of IS in glass screw tubes in an ultrasonic water bath for 2 × 2 h at a temperature not exceeding 50 °C, with periodic mixing using a vortex mixer. The extracts were separated, mixed and evaporated to dryness under a nitrogen stream at 35 °C. Reconstitution was performed by adding 300 µL of methanol. The obtained solutions were placed in 2 mL vials with inserts for analysis, and 3 µL was injected into the system.

### 2.4. Instrumentation

The analysis was performed using a liquid chromatography–triple quadrupole mass spectrometer (LC-MS/MS) 8060 system (Shimadzu, Kyoto, Japan) and a Shimadzu SP-C18 column (2.1 × 150 mm, 2.7 μm; Shimadzu, Kyoto, Japan). Separation was performed at 30 °C. The analysis was performed with a flow rate of 0.2 mL/min using a water gradient as solvent A and methanol as solvent B, starting at 15% of solvent B (0–1 min), raising to 95% of solvent B (1–16 min), and finally lowering back to 15% of solvent B (20 min). The quantitative and qualitative analyses of BPA were performed in multiple reaction monitoring (MRM) mode to track precursor–product ion transitions (ESI). The nebulizing gas flow rate was 2.5 L/min, the drying gas flow rate was 10 L/min, and the heating gas flow rate was 10 L/min. Interface, desolvation line (DL), and heat block temperatures were set to 300 °C, 200 °C, and 200 °C, respectively. The retention times and the monitoring ions (m/z) for each substance are presented in Table 2.

### 2.5. Method Validation

The method’s efficacy was evaluated using standard solutions of BPA (0, 6.25, 12.5, 25, 50, 100, and 250 ng/mL) prepared in methanol, with a linearity of 0.9955 (*n* = 4). Cat hair with no detectable levels of BPA or BPA levels close to the LOQ of the method was collected and used as blank samples. Blank samples spiked with known amounts of BPA were used to validate the method’s analytical parameters at concentrations of 0, 12.5, 25, 50, 100, 250, and 500 pg/mg. The linearity was found to be 0.9957 (*n* = 6). The limit of detection (LOD) and quantification (LOQ) were evaluated using the standard error and the slope of the calibration curves and calculated at 4.2 pg/mg and 12.7 pg/mg, respectively. The mean % recovery and accuracy values of the method were determined at 83.7% (*n* = 6) and 110.4% (*n* = 6), respectively, while the corresponding mean %RSD was 18.5% (*n* = 5) for the spiked levels of 12.5, 25, 50, 100, and 500 pg/mg of the hair. To ensure system suitability, a blank solvent, a standard solution and at least two spiked samples were analysed for each batch of ten authentic samples. Validation parameters of the method are summarised in Table 3.

### 2.6. Statistical Analysis

The statistical analysis was prepared using GraphPad Prism version 9.2.0 (GraphPad Software, San Diego, CA, USA). Descriptive statistics, including the minimum, 25th percentile, median, 75th percentile, maximum, arithmetic mean, standard deviation (SD), standard error of the mean (SEM), geometric mean, and geometric SD factor, were used to characterise BPA levels in all samples. Differences in levels of BPA between two groups of cats (between animals of various genders and between outdoor and indoor cats) were evaluated with a non-parametric Mann–Whitney test. Differences in BPA levels between three groups of animals (in cats of various ages and in cats with various body condition scores) were evaluated with a non-parametric Kruskal–Wallis test with post hoc Dunn’s test. The non-parametric tests were used because the data showed non-normality, as verified earlier with the Shapiro–Wilk test. Values below LOD were included in the statistical analysis as LOD/2 (2.1 pg/mg). Moreover, correlations between BPA concentrations and the weight of animals were evaluated with Spearman’s rank correlation coefficient with the following interpretation: value of coefficient factor (Rs) 0.00 to 0.19—a very weak correlation; 0.2 to 0.39—a weak correlation; 0.4 to 0.69—a moderate correlation; 0.7 to 0.89—a strong correlation; and 0.9 to 1.0—a very strong correlation. BPA concentrations lower than the LOD and LOQ were included in the statistics as LOD/2 and LOQ/2, respectively. Moreover, the correlation between BPA concentrations and the weight of animals was evaluated with the Pearson correlation coefficient with the following interpretation: value of coefficient factor: 0.7 to 1.0 (−0.7 to −1.0)—strong positive (negative) correlation; 0.4 to 0.7 (−0.4 to −0.7)—moderate positive (negative) correlation; and 0 to 0.4 (0 to −0.4)—weak positive (negative) correlation.

## 3. Results

During the present investigation, BPA levels above LOD were found in the majority of samples (97%; Table 4 and Table S1).

The extreme differences in BPA concentrations across hair samples were evident: the lowest concentration was below LOD, and the highest observed in this study was 955.4 pg/mg. The mean concentration of BPA (±SD) was 68.0 ± 145.2 pg/mg, and the median was 27.3 pg/mg. Data on the concentration of BPA in cat hair from the present study are summarised in Table 4.

**Figure 2:**
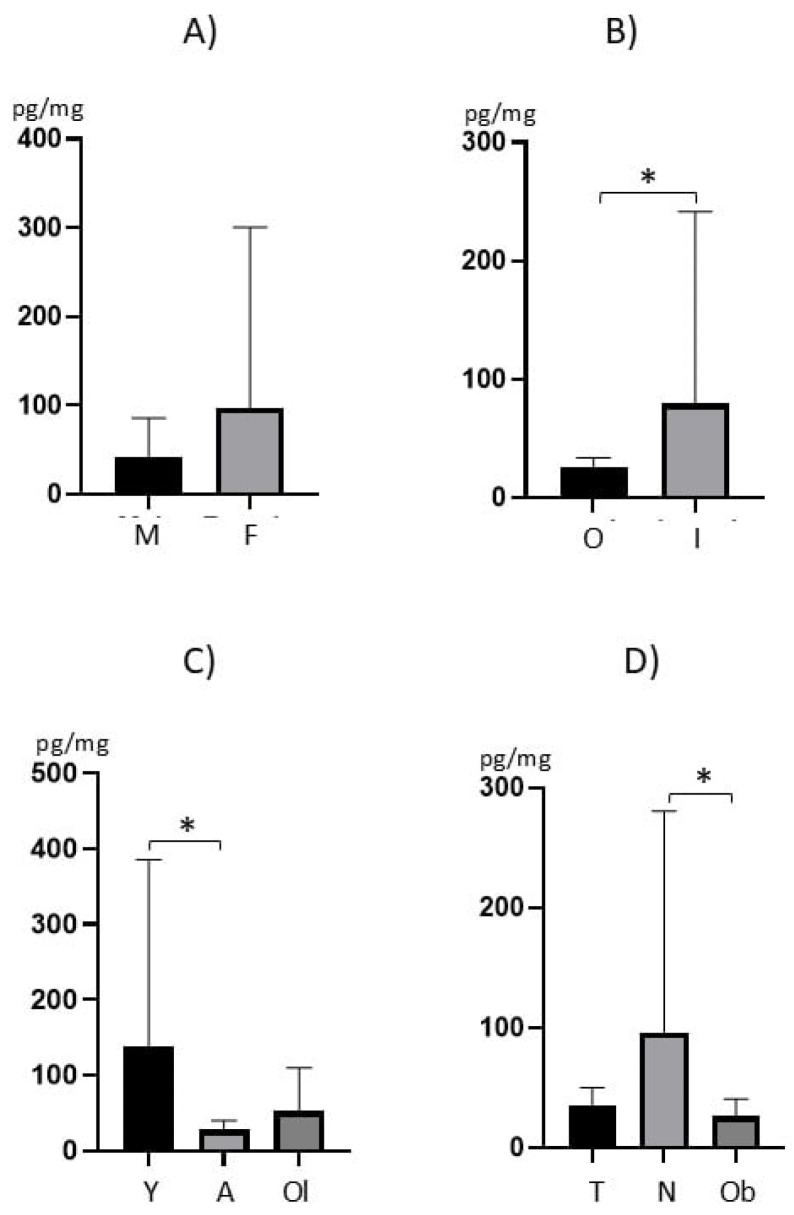
Mean concentration (±SD) of BPA in (**A**) males (M) and females (F); (**B**) outdoor (O) and indoor (I) cats; (**C**) young (Y), adult (A) and old (Ol) cats; (**D**) in too-thin (T), normal (N) and obese (Ob) cats. Statistically significant differences are indicated with asterisks.

In males (*n* = 37), the mean levels (±SD) of BPA amounted to 42.4 ± 43.9 pg/mg and the median was 28.9 pg/mg. In females (*n* = 33), these values were 96.7 ± 204.2 pg/mg and 24.6 pg/mg, respectively. Differences in BPA concentration levels between animals of various genders were not statistically significant (*p* = 0.9650) (Figure 2A).

During the present study, clear differences in BPA levels were found between outdoor and indoor cats. In outdoor cats (*n* = 15), the mean BPA level was 25.9 ± 8.1 pg/mg, and the median was 24.4 pg/mg. In indoor cats (*n* = 55), BPA concentrations were higher, with a mean of 79.5 ± 162.2 pg/mg and a median of 35.3 pg/mg. Differences in BPA concentration levels between outdoor and indoor cats were statistically significant (*p* = 0.0373) (Figure 2B).

Some differences in BPA concentration levels were also noted between animals of different ages (Figure 2C). In the young animals (≤2 years) (*n* = 21), the mean concentration of BPA was 137.1 ± 248.4 pg/mg, and the median was 58.0 pg/mg. In adult cats (from 2< to ≤6 years) (*n* = 28), BPA concentration levels were lower, with a mean of 27.9 ± 12.1 pg/mg and a median of 23.2 pg/mg. In turn, in old cats (over 6 years) (*n* = 21), the mean concentration of BPA amounted to 52.4 ± 57.6 pg/mg and the median was 35.3 pg/mg. Statistically significant differences in BPA levels were found between young and adult animals (*p* = 0.0071). Differences between young and old cats, as well as between adult and old cats, were not statistically significant (*p* = 0.6494 and *p* = 0.560, respectively).

**Figure 3:**
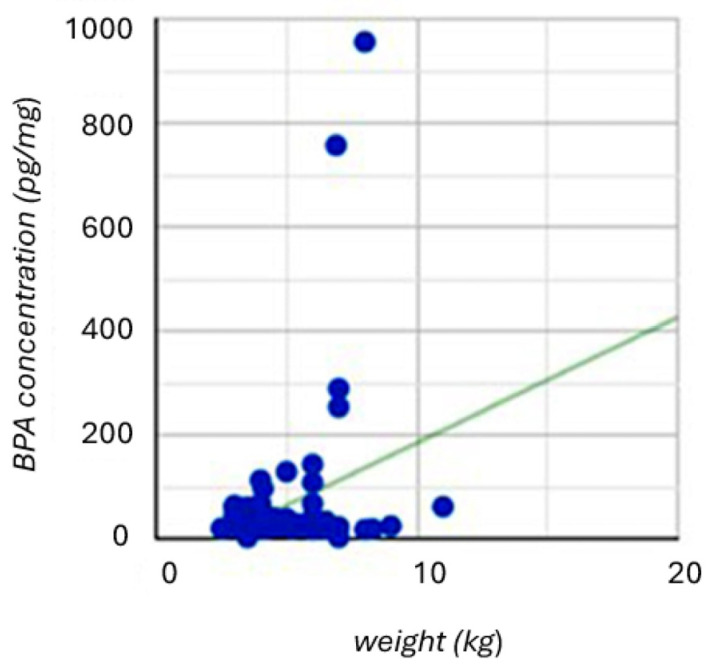
Correlation between BPA concentration levels in cat hair and body weight. A weak positive correlation was observed (Spearman’s rank correlation coefficient r = 0.272, *p* = 0.023, *n* = 70). The solid line represents the linear regression trend.

Some differences in BPA concentration were also observed depending on animal body condition score (Figure 2D). In cats with BCS 1–4 (*n* = 12), the mean concentration of BPA was 35.0 ± 15.1 pg/mg, and the median was 29.3 pg/mg. In cats with BCS 5 (*n* = 41), these values were 95.1 ± 185.4 pg/mg and 33.3 pg/mg, respectively. In turn, in cats with BCS of 6–9 (*n* = 17), the mean concentration of BPA was 25.8 ± 14.5 pg/mg, and the median was 22.4 pg/mg. Differences between animals with BCS of 5 and cats with BCS of 6–9 were statistically significant (*p* = 0.0190). Differences between cats with BCS of 1–4 and animals with BCs of 5, as well as between cats with BCS of 1–4 and cats with BCS of 6–9, were not statistically significant (*p* = 0.9999 and *p* = 0.2949, respectively). Moreover, it was found that BPA concentration in cat hair showed a weak positive correlation (r = 0.272) with cat weight, which was statistically significant (*p* = 0.023) (Figure 3).

## 4. Discussion

The results obtained clearly show that cats are frequently exposed to BPA. Such a situation is logical because cats, together with dogs, are species that live in close proximity to humans. For this reason, they are particularly exposed to anthropogenic pollutants. Commercial food, toys and animal cosmetics are also important sources of companion animal exposure to endocrine-disrupting chemicals, including BPA [40,41,48]. High exposure of cats and dogs to such substances has been confirmed by numerous works [35,47,49,50]. On the other hand, it should be noted that knowledge of cat exposure to BPA is extremely scarce and, to the authors’ knowledge, limited to only three previous investigations (Table 5).

Based on pharmacokinetic studies [31], it can be concluded that the BPA levels observed in cat hair in this study may be the result of relatively high animal exposure to this substance. However, a direct comparison of the present results with previous observations is not possible because clear discrepancies in BPA levels have been observed between urine and hair [51,52,53], which is due to the different nature of these matrices. BPA levels in urine, serum, and faecal samples analysed earlier are subject to rapid, short-term fluctuations. Pharmacokinetic studies have shown that BPA levels in the urine, serum and faeces increase rapidly within a few hours of exposure and decline again within one or two days [31,54]. In the case of hair, the situation is different. BPA accumulates in hair, and its levels in hair are not subject to rapid, short-term changes [30,31]. For this reason, hair is a suitable matrix for studies of long-term exposure lasting weeks or even months. Taking into account the length of the hair studied (0.7 cm–1.8 cm) and the fact that the hair growth rate in cats is approximately 0.8 to 1.3 cm/month [55,56,57], it can be concluded that the temporal window of BPA exposure assessed during the present investigation was about one month or a little over a month. Of course, this value is only approximate, as the rate of hair growth in cats is influenced by many factors, such as age, diet, season, hormonal activity, gender, and even the part of the body where hair grows [56,57]. The second difficulty in comparing these results with previous studies on BPA in cats is that the earlier studies were conducted in different regions of the world (Table 5), and interregional differences in BPA exposure are well documented [46,58].

It is interesting to compare the present results with the levels of BPA observed in human and other domestic animal hair (Table 6).

Due to interregional differences in exposure to BPA, the comparison of the present results with two previous studies on BPA levels in human [61] and dog hair [47], both conducted in the same city as this research, is especially interesting (Table 6). Analysing these studies, it can be concluded that BPA levels in cat hair, similarly to those in dog hair [47], are higher than those observed in humans living in the same area [61]. These observations are somewhat surprising, as human exposure to anthropogenic environmental pollutants (including BPA) is typically higher than that of animals. It is associated with many factors important to humans but not to animals, such as occupational exposure and the use of clothing, cars, and electronic equipment, among others [2,3]. Higher BPA levels in the hair of cats and dogs compared to the hair of people living in the same area strongly support thesis that pet animals are more exposed to BPA than humans, which may be connected with their size (greater exposure to house dust, which may contain BPA), relatively high levels of BPA in food or the presence of this compound in toys, bowls or care and training devices. Previous studies have clearly indicated the presence of BPA in cat and dog food (especially canned food) and toys [40,41,48], as well as a correlation between feeding with canned food and BPA levels in cat and dog organisms [34,64]. It should be pointed out that the use of BPA in the production of pet food packaging and other items intended for animals is not subject to any legal restrictions, unlike the production of items for people, especially for children [65]. This high exposure means that pets are considered good sentinel and indicator species which can warn humans about environmental pollution with various harmful substances [37]. On the other hand, the relatively high BPA levels observed in cats in this study may be due to interspecies differences in their metabolism. The metabolic processes of BPA in cats are poorly understood. Nevertheless, it is known that one of the main pathways of BPA metabolism is glucuronidation [66,67]. In turn, in cats, the gene encoding UDP-glucuronosyltransferase (UGT) 1A6, which is crucial for this reaction, is a non-functional pseudogene [68,69]. This pseudogenisation, resulting from an evolutionary loss-of-function mutation, reduces cats’ ability to metabolise BPA and other phenolic compounds [69], potentially leading to greater accumulation of these substances in the body. This is supported by previous studies that found that BPA concentrations in cat urine were 20 times higher than in dog urine [39]. However, BPA levels in this study were lower than those reported in the hair of dogs from the same city [47]. It should also be underlined that BPA levels in cat hair noted in the present study are lower than levels noted in the hair collected from humans and farm animals living in other regions of the world (Table 6). Moreover, because the UGT1A6 gene is a non-functional pseudogene across species [68,69] and differences in its activity among individual cats have not been confirmed, pseudogenisation of UGT1A6 is unlikely to explain the extreme differences in BPA levels reported in this study.

In the current study, no statistically significant differences in BPA levels were found between male and female cats. It should be underlined that the issue of the relationship between gender and the level of BPA in the body is not fully explained, and the results of previous studies on this subject are ambiguous. In studies on human exposure to BPA, some have reported higher BPA levels in men than in women [70], whereas others have reported the opposite [71]. Still, other studies conducted on humans from the same city or on humans living in the same household have shown no differences in BPA levels between men and women [72,73]. In humans, intragender differences in BPA levels are explained in two ways. Firstly, they may result from the different activities of sex hormones influencing the metabolism of BPA, and secondly, they may be connected with different lifestyles between men and women [70,74]. In animals, intragender differences in BPA levels are also not clear. In previous studies on cats and dogs [39,47], like in the present results, statistically significant intragender differences were absent in BPA levels, although studies on dogs [47] have reported higher BPA levels in females, as in the present study. In turn, in wild boars, BPA levels were statistically significantly higher in females than in males [33]. In animals, differences in BPA levels between males and females may result from different metabolism rates and hormonal activity [47].

In the present study, clear differences in BPA levels were found between strictly indoor cats and cats with outdoor access. Higher levels of BPA in the serum of indoor cats have also been shown in previous studies [34]. These observations strongly suggest that the domestic environment is heavily contaminated with BPA and that cats living in this environment are constantly highly exposed to its effects. In light of previous studies, dust is one of the most important sources of BPA in indoor environments [75]. However, higher indoor cat exposure to BPA may also be connected with long-term contact with home appliances, carpets, furniture, etc.

The observations on correlations between BPA levels and animal age are less clear. In the present study, higher levels of BPA were found in animals aged 2 years or younger. Previous data on BPA in cats and dogs are inconclusive. Some studies have not found age-dependent statistically significant differences in BPA levels [39,47]. Other studies have reported such differences, but knowledge of them is limited to cats over 7 years old [34]. Previous studies on BPA levels in humans have reported different results. Some authors have reported higher levels in younger people and children [71,76], whereas others have reported higher levels in older humans [25]. Age-dependent differences in BPA levels are explained by various factors, including differences in hormonal activity, metabolic rate, physical activity, the amount and type of food consumed by individuals of different ages, as well as developmental and maturation processes in young organisms and ageing processes in older ones [25,76]. Physiological processes within the gastrointestinal tract also seem important. It has been shown that, in young organisms, due to not fully developed digestive and enzymatic functions, BPA metabolism is less efficient, leading to higher levels of this substance in the body [77]. On the one hand, this could explain the higher levels observed in young cats in the present experiment. On the other hand, the digestive tract is the least efficient at metabolising BPA in newborns [77], and in this study, the youngest cat group included older animals. Moreover, the metabolism of BPA is poorly understood in cats, and it is unknown whether it is slower in young animals.

Interestingly, during the present study, BPA levels in animals with normal body condition score (BCS 5) were statistically significantly higher than in cats with obesity (BCS 6–9). This result is surprising because BPA is a substance that is considered to be one of the factors causing metabolic disorders and, as a result, obesity [78]. It is well established that BPA affects adipose tissue and alters the production and secretion of adipokines, thereby playing an important role in the pathogenesis of obesity [79,80,81,82]. Additionally, numerous studies have shown a positive correlation between BPA exposure and the risk of obesity in humans [78,81,83,84]. The results obtained in the present study are difficult to explain, as many aspects of BPA metabolism in cats and its accumulation in hair remain poorly understood. The explanation of this phenomenon remains speculative. However, lower BPA levels in obese cats (BCS 6–9) may be associated with obesity negatively affecting hair growth rate. Hormonal disturbances, chronic inflammatory processes, and insulin resistance that accompany obesity disrupt the hair follicle cycle and deplete stem cells, leading to reduced growth, thinning, and hair loss [85,86]. Slower hair growth means a shorter temporal window of BPA exposure, resulting in lower levels of this substance. Moreover, it is known that BPA, due to lipophilic nature, has a high affinity for adipose tissue, where it can accumulate in significant amounts [87,88]. Previous research has demonstrated that lipophilic environmental pollutants, including BPA, may accumulate preferentially in adipose tissue, potentially reducing their concentration in other matrices such as blood, urine, and hair [87]. This sequestration effect could partially explain the lower BPA levels observed in hair samples from obese cats in the present study, because a significant amount of subcutaneous fat tissue may hinder the penetration of BPA into the hair follicles. Another reason for the lower level of BPA in the hair of obese cats (BCS 6–9) may be related to the behaviour of such animals, which, for example, are reluctant to use toys and are less active, which is associated with a lower risk of exposure to BPA. It is also possible that owners of obese cats introduce dietary foods that may be less contaminated with BPA than standard commercial foods, or they may limit the amount of food given to the animal, thereby reducing the animal’s exposure to BPA. On the other hand, observed differences in BPA levels may be linked to other unidentified local environmental factors in this study. This is all the more likely because extreme differences in BPA levels between individual animals (much more visible than those noted in the hair of other species—Table 6) have been found in the present study. In turn, a weak, positive, statistically significant correlation between BPA concentration and cat weight suggests BPA’s obesogenic activity.

Analysing the obtained results, a certain discrepancy can be noticed. Namely, obese cats (BCS 6–9) show significantly lower BPA, whereas body weight showed a weak positive correlation with BPA concentration. This discrepancy may result from the fact that BCS scale represents a categorical assessment of body condition based on visual and palpable characteristics, whereas absolute body weight is a continuous metric that may not directly correlate with adiposity in cats of different body sizes and breeds. A small-framed cat with normal BCS and a large-framed obese cat may have similar body weights. Therefore, the positive correlation between body weight and BPA levels may reflect overall body size and exposure surface area rather than adiposity per se. This could explain the apparent discrepancy between lower BPA levels in high-BCS cats and the positive weight–BPA correlation observed in this study. Nevertheless, this aspect requires further investigation.

The question arises whether the levels of BPA observed in hair in this study may result from exposure that could negatively affect animal health. Given current knowledge, it is difficult to answer this question. It is well established that the metabolism of BPA and its associated adverse effects vary across animal species [89]. Knowledge of BPA’s impact on cat health is extremely limited. Few studies on this issue have described that BPA inhibits spontaneous contractions of the cat uterus, which can lead to infertility [42] and negatively affects the process of seeing and neuronal transmission [43,90,91]. Correlations between the degree of cat exposure to BPA and red blood cell count, haemoglobin levels, calcium concentration, and bilirubin levels in blood serum have also been reported [34]. On the other hand, in cats, the influence of BPA on thyroid function has not been observed [34], although such an effect is well established in other species [92,93]. Because the exact mechanisms of BPA metabolism in cats are not known, it is difficult to determine the degree of exposure that caused the observed hair levels of BPA in this study. Based on pharmacokinetic studies in rats, it can be assumed that the BPA levels observed in this study reflect relatively high exposure [31]. Moreover, it is well established that even low doses of BPA can hurt human and animal health, affecting various organs and systems [94,95,96]. It is also known that companion animals are exposed to many environmental pollutants, which often have a synergistic effect. Li and Kannan [35] have described 121 environmental chemicals in cat and dog urine and faeces. The most important of them, apart from BPA, were other bisphenols, phthalates, parabens and pesticides [35]. In the case of synergistic interactions among multiple pollutants, even low concentrations of individual compounds can be harmful. Taking this into account, it can be assumed that the degree of exposure to BPA resulting in the observed levels in this experiment may negatively affect the health of these animals. Nevertheless, clarification of all aspects related to cats’ exposure to BPA and its negative impact on their health requires further comprehensive environmental, toxicological, and clinical studies.

The most serious limitations of this research should also be mentioned. The most important of them is the lack of identification of environmental factors that increase the risk of BPA exposure in the individual animals included in the study. It is known that cat exposure to BPA may depend on numerous various factors which, in addition to the diet, may include, among others, the type of bowls from which the animals are fed, household cleaning product usage, the presence of smokers in the household and type of furnishing materials (Figure 1). Understanding these factors is important from the point of view of veterinary toxicology, and the only way to determine them is to conduct detailed surveys among cat owners. Therefore, the absence of such surveys is a limitation of this research. On the other hand, pet owners are very reluctant to complete surveys or give not entirely honest answers. For this reason, survey results can often be unreliable. The second limitation of this study is a lack of data on animal health parameters (liver enzymes, thyroid hormones, reproductive markers, etc.). Generally, this study included clinically healthy animals. Therefore, the collection of blood samples for such analyses would require the ethics committee to provide consent for animal research. Moreover, animal owners are reluctant to consent to medical procedures on healthy animals, even as part of veterinary preventive care. Including detailed surveys and blood tests in the research could drastically reduce the number of owners who would consent to their animal participating. The third limitation of this research is the relatively small sample sizes in some animal subgroups (e.g., outdoor cats or cats that are excessively thin). This situation results from the fact that in Poland, there are generally few cats with owners living in cities, and those that do are too thin and/or lack access to the outdoors. Despite these limitations, this study enriches the knowledge about cats’ exposure to BPA.

## 5. Conclusions

This study is the first to provide a description of BPA levels in cat hair. The obtained results have clearly demonstrated the widespread occurrence of BPA and its relatively high levels in cat hair samples. Such observations strongly suggest that long-term exposure of cats to BPA is significant. Moreover, visible differences in BPA levels across particular hair samples suggest that cats’ exposure to BPA may depend on local factors. It has also been shown that strictly indoor cats are more exposed to BPA than cats with outdoor access. It suggests that the home environment is heavily contaminated with BPA. Some differences in BPA levels among animals of various ages and body condition scores suggest that these factors may also affect BPA levels in cats. During the study, no statistically significant differences in BPA levels were found between males and females. Relatively high levels of BPA in hair suggest that exposure to this compound may negatively affect cats’ health. However, current knowledge of BPA metabolism in cats and its impact on the feline organism is very limited. Therefore, further comprehensive environmental, toxicological and clinical studies are required to fully elucidate all aspects of cat exposure to BPA and its associated health effects.

## Figures and Tables

**Table 1:** Characterisation of Olsztyn.

Coordinates	53°46′40″ N 20°28′45″ E
Human population	166,392
Area (km^2^)	88.32
Population density (persons/km^2^)	1884
Annual amount of municipal waste (tons) in 2024	50,635.45
Annual amount of plastic waste (tons) in 2024	3656.39
Main industries	rubber (tyre production), furniture, wood, metal and food industries
Number of cars registered in Olsztyn (data from July 2025)	145,000

Data according to the Polish Central Statistical Office (https://stat.gov.pl/).

**Table 2:** Retention times and monitoring m/z ions used in the present study.

	Rt (min)	Precursor m/z	Product Ion m/z	Dwell Time	Q1 (V)	CE	Q3 (V)
Bisphenol A	11.17	227.1	212.1	20	12	18	14
		227.1	133.1	20	26	25	27
Phenobarbital d5 (IS)	11.96	236.1	85.0	20	26	12	11
		236.1	42.0	20	13	17	15

**Table 3:** Validation parameters of the method used.

	BPA	No. of Repetition (Repet.)	Concentration Range
LOD (pg/mg)	4.2	-	-
LOQ (pg/mg)	12.7	-	-
% recovery	83.7	6	12.5–500 pg/mg
% accuracy	110.4	6	12.5–500 pg/mg
Inter day precision (%RSD)	18.5	5	12.5–500 pg/mg
r^2^ standard solutions	0.9955	4	0–250 ng/mL
r^2^ spiked samples	0.9957	6	0–500 pg/mg

**Table 4:** Concentration level (pg/mg) and frequency (%) of detection of BPA in cat hair samples (*n*  =  70)—cumulative data.

Minimum	<LOD
25% percentile	22.2
Median	27.3
75% percentile	53.0
Maximum	955.4
Arithmetic mean	68.0
Standard deviation (SD)	145.2
Standard error of mean (SEM)	17.4
Geometric mean	35.6
Geometric SD factor	2.6
% samples > LOD (%)	97
% samples > LOQ (%)	97

**Table 5:** Mean levels of BPA (±SD) in cats in the light of previous studies (in liquid matrices ng/mL; in solid matrices pg/mg); n—number of samples.

Country/Region	n	Matrix	BPA Concentration	Reference
Czech Republic	69	blood serum	1.06 ± 0.908	[34]
USA/NY	50	urine	22.3 ± 155	[39]
USA/NY	46	urine	0.59 ± 0.39	[35]
USA/NY	25	faeces	29.2 ± 34.3	[35]
Poland	70	hair	67.98 ± 145.2	This study

**Table 6:** BPA levels in hair samples according to previous studies.

Country	n	BPA Levels (Median) (pg/mg)	Reference
humans			
Belgium	114	<LOQ − 587 (46.6)	[29]
China	204	5.47–596 (34.9)	[59]
Greece	100	9.6–650.3 (69.9)	[28]
Luxembourg	264	62.34–35,856.1 (133.6)	[60]
Poland	25	<LOD − 52.9 (17.7)	[61]
Spain	42	24.4–1427 (195.1)	[25]
dogs			
Poland	30	<LOD − 436 (47.35)	[47]
sheep			
Poland	50	75.7–472 (135.5)	[62]
Kyrgyzstan	50	46.9–502 ng/g (100.5)	[62]
dairy cows			
Kyrgyzstan	48	<LOD − 89.1 (<LOD)	[63]
cats			
Poland	70	<LOD − 955.4 (27.3)	This study

n—number of samples; LOQ—limit of quantification; LOD—limit of detection.

## Data Availability

All data are available in the manuscript and Supplementary Materials.

## Supplementary Materials

The following supporting information can be downloaded at https://www.mdpi.com/article/10.3390/ani16040567/s1, Table S1: Characterization of cats included into the study and BPA levels.
