# Supplementary material for: Evaluation of Cat Exposure to Bisphenol A (BPA) Using Hair Sample Analysis
Source: Animals (Basel). 2026 Feb 12;16(4):567. doi: 10.3390/ani16040567 (PMC12937472; doi:10.3390/ani16040567)
Supplement: Supplementary file 1 [file animals-16-00567-s001.zip › animals-4119683-supplementary.pdf]

Supplementary materials to

Evaluation of Cat Exposure to Bisphenol A (BPA) Using Hair Sample Analysis

Slawomir Gonkowski [1,*,†], Manolis Tzatzarakis [2,†], Elena Vakonaki [2], Thomas Lamprakis [2] and Krystyna Makowska [3,*]

[1] Department of Clinical Physiology, Faculty of Veterinary Medicine, University of Warmia and Mazury in Olsztyn, Oczapowskiego 13, 10-719 Olsztyn, Poland

[2] Laboratory of Toxicology, School of Medicine, University of Crete, 71003 Heraklion, Crete, Greece; tzatzarakis@uoc.gr (M.T.); evakonaki@gmail.com (E.V.); lamprakist@gmail.com (T.L.)

[3] Department of Clinical Diagnostics, Faculty of Veterinary Medicine, University of Warmia and Mazury, Oczapowskiego 14, 10-719 Olsztyn, Poland

* Correspondence: slawomir.gonkowski@uwm.edu.pl (S.G.); krystyna.makowska@uwm.edu.pl (K.M.)
† These authors contributed equally to this work.

Table S1. Characterization of cats included into the study and BPA levels

Animal no.	Gender	Age (years)	Outdoor (O) or indoor (I) cat	BCS scale	BPA levels (pg/mg)	
					Mean (n=2)	±SD (n=2)
1	male	7	I	3	35.3	1.6
2	female	7	I	5	96.8	13.7
3	female	1	I	5	65.7	9.8
4	male	8	I	5	40.8	3.2
5	female	7	I	5	112.9	1.0
6	female	1	I	5	19.9	2.7
7	female	5	I	5	60.6	3.4
8	male	13	I	5	23.5	3.0
9	male	12	I	4	41.2	4.5
10	male	7	I	6	35.8	7.0
11	female	1	I	4	64.2	8.5
12	male	1	I	5	107.9	29.9
13	female	8	O	5	36.4	0.9
14	female	14	I	5	21.3	1.6
15	male	5	I	5	37.9	1.3
16	male	5	I	5	39.1	1.5
17	male	1,5	I	3	59.9	15.0
18	male	1	O	5	18.5	0.8
19	male	5,5	O	6	33.8	<0.1
20	male	8	I	7	24.2	-

21	male	9	I	6	37.8	10.5
22	female	10	I	5	23.4	2.5
23	female	6	I	7	22.4	0.9
24	female	1,5	I	5	955.4	107.4
25	male	1	O	4	24.9	3.7
26	male	10	I	5	22.3	1.1
27	male	4	I	3	23.5	2.3
28	female	1,5	I	5	288.6	25.7
29	male	7	I	5	142.9	11.0
30	male	6	I	7	19.6	0.8
31	female	1	O	3	22.9	2.5
32	female	1,5	I	5	58.0	5.6
33	female	5	I	7	21.2	2.7
34	male	5	I	5	22.9	3.9
35	male	4	I	5	51.3	21.0
36	female	4	I	5	33.3	5.5
37	male	15	I	5	253.5	5.6
38	male	1	I	5	25.4	0.7
39	male	5	I	6	38.1	0.1
40	female	2	O	4	29.7	3.8
41	female	1,5	I	5	20.1	2.6
42	Male	6	I	5	21.4	2.9
43	Male	4	I	5	25.7	3.5
44	female	11	I	7	<LOD	-
45	male	5	I	5	24.5	3.8
46	male	4	O	6	17.7	0.5
47	male	5	I	7	<LOD	-
48	female	10	I	3	19.9	2.0
49	male	14	I	7	18.1	4.4
50	female	2,5	O	5	18.2	1.2
51	male	6	I	4	28.9	5.1
52	male	2	I	5	42.3	13.1
53	male	2	I	5	32.5	11.5
54	female	2,5	O	5	22.3	3.4
55	female	5	I	5	19.4	1.6
56	female	5	I	7	40.4	6.0
57	male	7	O	5	25.7	2.9
58	female	2,5	O	3	47.5	10.1
59	female	2	I	5	67.9	40.1
60	male	6	O	7	19.8	0.3
61	female	5	O	5	24.4	5.5
62	female	5	I	5	22.2	6.4
63	female	2	I	5	756.6	96.7
64	female	1	I	5	129.3	6.9
65	male	7	I	6	61.5	4.5

66	male	4	O	5	22.5	6.5
67	female	5	I	6	19.3	5.7
68	female	7	O	6	24.6	10.7
69	male	2	I	5	66.9	24.3
70	female	2	I	4	22.2	5.7

LOD- limit of detection
